# A new paradigm for cancer immunotherapy: Orchestrating type 1 and type 2 immunity for curative response

**DOI:** 10.1002/ctm2.70154

**Published:** 2024-12-31

**Authors:** Bing Feng, Li Tang

**Affiliations:** ^1^ Institute of Bioengineering École Polytechnique Fédérale de Lausanne (EPFL) Lausanne Switzerland; ^2^ Institute of Materials Science & Engineering EPFL Lausanne Switzerland; ^3^ Hangzhou Institute of Medicine Chinese Academy of Sciences Hangzhou China

**Keywords:** cancer immunotherapy, curative response, Type 2 immunity

1

The immune system serves a vital function in safeguarding the body against external pathogens and eradicating cancerous cells. Depending on the nature of pathogens encountered, the immune system orchestrates distinct immunological strategies: type 1 immunity primarily targets intracellular pathogens, including viruses and certain bacteria; type 2 immunity is adept at combating parasites within mucosal tissues and the dermis; while type 3 immunity is instrumental in the clearance of extracellular fungi and bacteria.^1^ Nevertheless, the mechanisms by which the immune system influences cancer initiation, progression, and therapeutic intervention remain partially elucidated. Traditionally, type 1 immunity has been regarded as the foremost defence against cancers, and current immunotherapeutic approaches including immune checkpoint blockade (ICB)^2^ and chimeric antigen receptor T‐cell (CAR‐T) therapy^3^ are crafted to activate or enhance type 1 immune responses against cancer. Despite the clinical success, the curative response of type 1‐centric immunotherapies has been limited, frequently resulting in a low patient response rate or high relapse rate with diminished durability of response.

CAR‐T therapy targeting CD19 has exhibited exceptional initial response rates in various malignancies derived from B‐cell lineages; however, relapse remains a formidable challenge. To elucidate the underlying factors contributing to relapse, we conducted a comprehensive single‐cell multi‐omics analysis of over one million pre‐infusion CAR‐T cells sourced from 82 pediatric patients with B‐acute lymphoblastic leukaemia (B‐ALL) and six healthy donors.^4^ The patients were participants in two of the pioneering global clinical trials for pediatric B‐ALL (NCT01626495 and NCT02906371), with a follow‐up duration extending beyond ten years. While some patients experienced relapse within two years post‐treatment, others achieved remarkable long‐term cancer‐free survival, enduring for up to eight years, thereby being considered cured of their malignancy. Clustering analysis revealed that the CAR‐T cell products from patients who were cured demonstrated a significantly higher proportion of CAR‐T cells characterized by type 2 immune signatures (type 2 high CAR‐T cells); conversely, no noteworthy differences were observed in the proportion of CAR‐T cells exhibiting type 1 immune signatures.^4^ Furthermore, preclinical experiments indicated that the proliferative capacity of type 2 high CAR‐T cells surpassed that of type 2 low CAR‐T cells by more than tenfold, indicative of their superior memory characteristics and diminished signs of exhaustion.^4^ In a recurrent leukaemia model, type 2 high CAR‐T cells effectively mitigated leukaemia relapse.^4^ These findings underscore the pivotal and previously underappreciated role of type 2 immunity in fostering the sustained efficacy of CAR‐T therapy against haematologic malignancies (Figure 1A).

**FIGURE 1:**
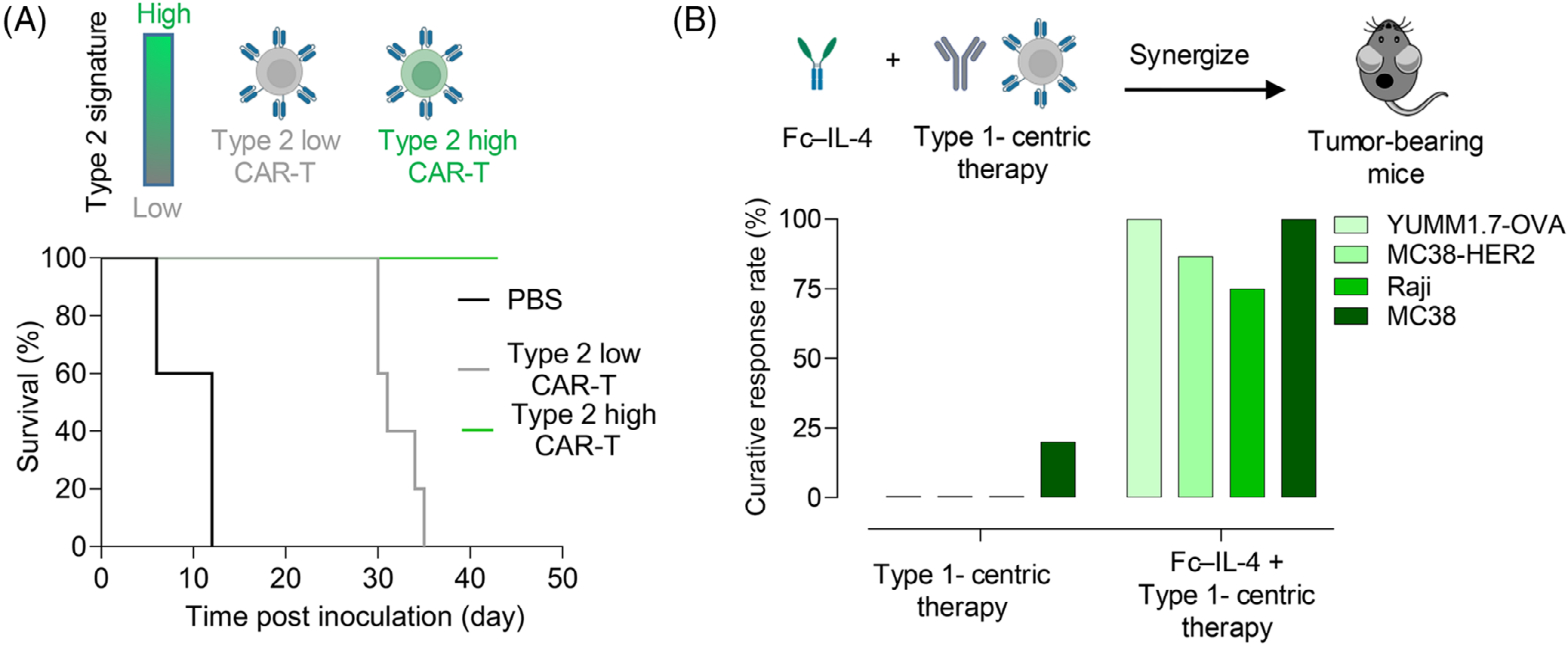
Type 2 immunity shows promise in enhancing cancer immunotherapy in mouse models. (A) Type 2 high chimeric antigen receptor T (CAR‐T) cells prevented leukaemia relapse and prolonged mouse survival. (B) Type 2 cytokine interleukin (IL)‐4 with an Fc fragment (Fc–IL‐4) synergized with type 1‐centric cancer immunotherapy to eliminate several solid tumours in mouse models.

Compared to hematologic malignancies, solid tumours pose an even more formidable challenge for immunotherapy due to their intricate and immunosuppressive tumour microenvironments (TME). The quality and functionality of CD8^+^ T cells—the principal cytotoxic effectors—are crucial determinants of the success of immunotherapeutic interventions in solid tumours. However, prolonged antigen exposure within the TME frequently drives T cells towards exhaustion, leading to diminished effector functions and compromised therapeutic persistence.^5^ Notably, the type 1 immunity cytokine interferon‐γ has been associated with the induction of T‐cell exhaustion and the selective depletion of tumour‐specific T cells,^6^ suggesting that excessive type 1 immune responses may paradoxically undermine long‐term antitumor efficacy.^7^ Given the regulatory role of type 2 immunity in modulating type 1 response and the sustained efficacy of type 2 high CAR‐T cells against hematologic malignancies, we hypothesized that type 2 immune factors could alleviate T‐cell exhaustion and enhance their functionality against solid tumours. To test this hypothesis, we engineered a fusion protein combining the type 2 cytokine IL‐4 with an Fc fragment (Fc–IL‐4) and assessed its effects on tumor‐infiltrating CD8^+^ T cells^8^ (Figure 1B). Remarkably, Fc–IL‐4 selectively enriched terminally exhausted CD8^+^ T (PD‐1^+^TIM‐3^+^TCF‐1^−^ and CD8^+^ T_TE_) cells, significantly augmenting their cytotoxicity and effector functions. Mechanistically, Fc–IL‐4 acted directly on CD8^+^ T_TE_ cells, which exhibited elevated expression of IL‐4 receptor α (IL‐4Rα) compared to their progenitors, thereby activating the signal transducer and activator of transcription 6 (STAT6) and mammalian target of rapamycin (mTOR) signalling pathways. This activation profoundly enhanced the glycolytic metabolism of CD8^+^ T_TE_ cells and upregulated a suite of glycolytic enzymes, particularly lactate dehydrogenase A (LDHA), thereby revitalizing the functionality of these T cells.

Subsequently, we assessed the therapeutic potential of combining Fc–IL‐4 with type 1‐centric cancer immunotherapies, including CAR‐T and ICB, across various solid tumour models. The results revealed that the combination therapies exhibited robust antitumor efficacy in multiple syngeneic and xenograft tumour models and elicited enduring immune memory effects. For instance, the administration of Fc–IL‐4 alongside OT1 T‐cell therapy achieved complete tumour clearance (100%) in the YUMM1.7‐OVA melanoma model. In the MC38‐HER2 colon cancer model, the combination of Fc–IL‐4 and HER2‐CAR‐T therapy resulted in 87% tumour eradication. In the Raji lymphoma model, pairing Fc–IL‐4 with CD19‐CAR‐T therapy led to 75% tumour clearance. Furthermore, combining Fc–IL‐4 and ICB resulted in a complete tumour clearance (100%) in the MC38 colon cancer model. Remarkably, all mice achieving tumour clearance demonstrated resistance to subsequent tumour rechallenge, underscoring the induction of robust and long‐lasting immune memory. As a representative type 2 immunity factor, Fc–IL‐4 not only revitalizes exhausted T cells but also complements type 1 immunity‐centric strategies, facilitating durable antitumor responses (Figure 1B).

We previously found that, as another cytokine associated with type 2 immunity, the interleukin‐10 (IL‐10)–Fc fusion protein can enhance the proliferative capacity and effector function of CD8^+^ T_TE_ cells through metabolic reprogramming.^9^ Additionally, CAR T cells can be metabolically optimized by engineering them to secrete IL‐10, facilitating the durable clearance of solid tumours and metastases.^10^ The IL‐10‐secreting anti‐CD19 CAR‐T cells are currently being tested in several ongoing first‐in‐human investigator‐initiated trials for the treatment of relapsed or refractory diffuse large B‐cell lymphoma or B‐ALL (Figure 2A).

**FIGURE 2:**
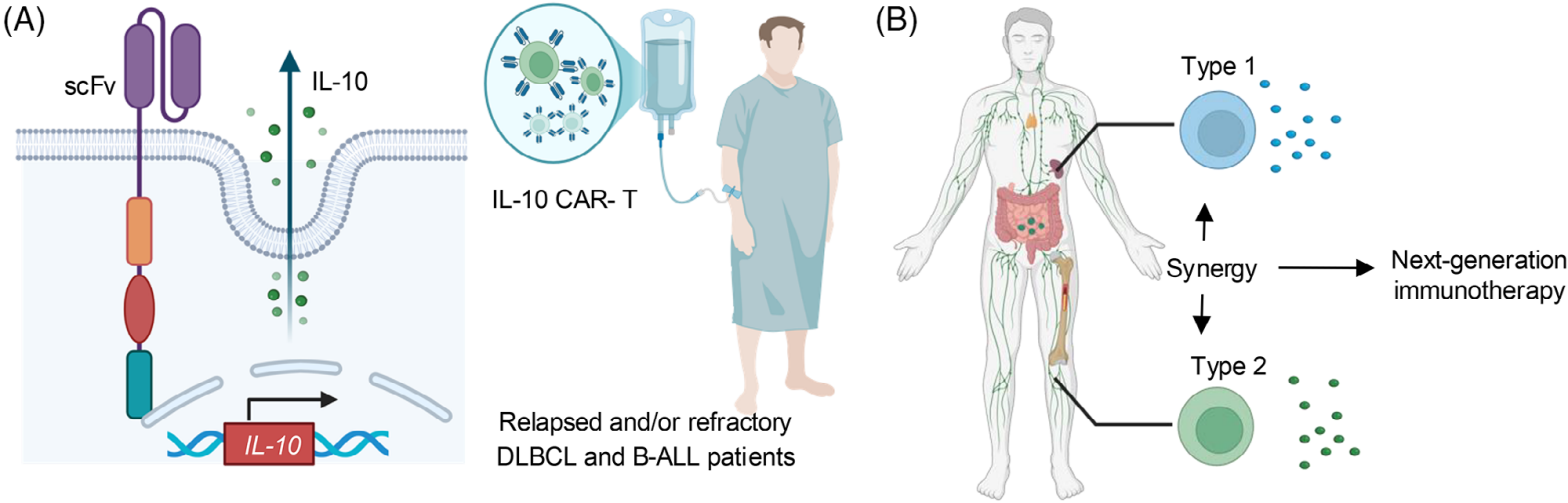
Orchestrating type 1 and type 2 immunity for next‐generation cancer immunotherapy. (A) The interleukin (IL)‐10‐secreting anti‐CD19 chimeric antigen receptor T (CAR‐T) cells are currently being tested in several clinical trials for the treatment of relapsed or refractory diffuse large B‐cell lymphoma (DLBCL) or B‐acute lymphoblastic leukaemia (B‐ALL). (B) Orchestrating type 1 and type 2 immunity to advance next‐generation cancer immunotherapy.

Collectively, these findings underscore the potential of type 2 immunity, particularly type 2 cytokine Fc–IL‐4, to advance current immunotherapies including CAR‐T and ICB therapies, which are centric on type 1 immunity. The investigation of type 2 immunity in cancer therapy and its synergy with type 1 immunity opens up a new paradigm for cancer immunotherapy in the clinic (Figure 2B).

## CONFLICT OF INTEREST STATEMENT

Li Tang is a co‐founder, shareholder, and advisor for Leman Biotech. The interests of Li Tang were reviewed and managed by EPFL. The remaining authors declare no competing interests.

## References

[1] Dupage M , Bluestone JA . Harnessing the plasticity of CD4+ T cells to treat immune‐mediated disease. Nat Rev Immunol. 2016;16:149‐163. 26875830 10.1038/nri.2015.18

[2] Wei SC , Levine JH , Cogdill AP , et al. Distinct cellular mechanisms underlie anti‐CTLA‐4 and anti‐PD‐1 checkpoint blockade. Cell. 2017;170:1120‐1133. 28803728 10.1016/j.cell.2017.07.024 PMC5591072

[3] Lee EHJ , Murad JP , Christian L , et al. Antigen‐dependent IL‐12 signaling in CAR T cells promotes regional to systemic disease targeting. Nat Commun. 2023;14:4737. 37550294 10.1038/s41467-023-40115-1 PMC10406808

[4] Bai Z , Feng B , McClory SE , et al. Single‐cell CAR T atlas reveals type 2 function in 8‐year leukaemia remission. Nature. 2024;634:702‐711. 39322664 10.1038/s41586-024-07762-w PMC11485231

[5] Blank CU , Haining WN , Held W , et al. Defining ‘T cell exhaustion’. Nat Rev Immunol. 2019;19:665‐674. 31570879 10.1038/s41577-019-0221-9 PMC7286441

[6] Mazet JM , Mahale JN , Tong O , et al. IFNγ signaling in cytotoxic T cells restricts anti‐tumor responses by inhibiting the maintenance and diversity of intra‐tumoral stem‐like T cells. Nat Commun. 2023;14:1‐21. 36658158 10.1038/s41467-023-35948-9 PMC9852295

[7] Gocher AM , Workman CJ , Vignali DAA . Interferon‐γ: teammate or opponent in the tumour microenvironment? Nat Rev Immunol. 2022;22:158‐172. 34155388 10.1038/s41577-021-00566-3 PMC8688586

[8] Feng B , Bai Z , Zhou X , et al. The type 2 cytokine Fc–IL‐4 revitalizes exhausted CD8+ T cells against cancer. Nature. 2024;634:712‐720. 39322665 10.1038/s41586-024-07962-4 PMC11485240

[9] Guo Y , Xie YQ , Gao M , et al. Metabolic reprogramming of terminally exhausted CD8+ T cells by IL‐10 enhances anti‐tumor immunity. Nat Immunol. 2021;22:746‐756. 34031618 10.1038/s41590-021-00940-2 PMC7610876

[10] Zhao Y , Chen J , Andreatta M , et al. IL‐10‐expressing CAR T cells resist dysfunction and mediate durable clearance of solid tumors and metastases. Nat Biotech. 2024;42:1693‐1704. 10.1038/s41587-023-02060-8 38168996

